# “Infectious Supercarelessness” in Discussing Antibiotic-Resistant Bacteria

**DOI:** 10.20411/pai.v1i2.160

**Published:** 2016-12-15

**Authors:** Neil S. Greenspan

**Affiliations:** Case Western Reserve University, Cleveland, Ohio

**Keywords:** bacterial pathogen, antibiotic resistance, fitness, virulence, transmissibility, resistance to immunity, evolution, colistin, *Escherichia coli*, *Mycobacterium tuberculosis*, multidrug resistance (MDR), extreme drug resistance (XDR), *Clostridium difficile*, fecal transplantation, vancomycin

## Abstract

Many bacterial pathogens are exhibiting resistance to increasing numbers of antibiotics making it much more challenging to treat the infections caused by these microbes. In many reports in the media and perhaps even in discussions among physicians and biomedical scientists, these bacteria are frequently referred to as “bugs” with the prefix “super” appended. This terminology has a high potential to elicit unjustified inferences and fails to highlight the broader evolutionary context. Understanding the full range of biological and evolutionary factors that influence the spread and outcomes of infections is critical to formulating effective individual therapies and public health interventions. Therefore, more accurate terminology should be used to refer these multidrug-resistant bacteria.

## A CONCERNING TREND TOWARDS GREATER ANTIBIOTIC RESISTANCE

In early 2016, there was a report [1] from infectious disease specialists at the Walter Reed National Military Medical Center of a patient infected with an *E. coli* strain resistant to colistin. This antibiotic has often been used to treat bacterial infections that are resistant to most other antibiotics and has therefore been referred to as a last resort treatment for *E. coli*. As has happened before with similar investigations, the publication of McGann *et al* triggered much coverage in the popular media [2, 3, 4] along with the reflexive references to so-called bugs that are to be regarded as super. Henceforth, I will refer to these bacterial pathogens as putatively super bacteria or microbes or, as described below, with other more directly descriptive terms.

## UNSATISFACTORY TERMINOLOGY

The key concept I wish to convey is that focusing solely on antibiotic resistance when evaluating the magnitude of a threat from a particular pathogen produces a potentially misleading analysis. In making the preceding point, I am in no way diminishing the magnitude of the threat posed by bacterial pathogens that exhibit resistance to multiple or, more ominously, all relevant antibiotics. On the contrary, while acknowledging the importance of this issue for individual patients and populations alike, I am suggesting that predicting the future trajectory of pathogen spread and fashioning better therapeutic strategies will both benefit from an awareness of the broader evolutionary context characterizing host-pathogen relationships.

The prefix “super” distracts and detracts from this broader perspective. This widely used modifier is so familiar to members of the general public as well as physicians and biomedical scientists, that it persuades those encountering it to believe that they know precisely what it means. Unfortunately, in the present medical context this assumption is likely to be incorrect for many of those hearing or reading about putatively super bacterial infectious agents. For example, this terminology might be interpreted to suggest that the highly drug-resistant bacteria are able to outcompete less resistant bacteria of the same species or spread more readily from person to person.

## THE RELEVANCE OF THE EVOLUTIONARY BIOLOGY OF PATHOGENS

In general, however, genetic elements mediating resistance for one or more antibiotics will not necessarily or simultaneously increase overall fitness (in the absence of antibiotics), or enhance virulence, transmissibility, or resistance to immune mechanisms [5, 6, 7]. While modification of such traits can result from further mutation and selection (i.e. further evolution) [8], there are generally trade-offs associated with modified phenotypes created via genetic alterations that will reveal themselves in one medically relevant circumstance or another.

## THE EXAMPLE OF *C. DIFFICILE* ENTEROCOLITIS

A particularly enlightening and dramatic illustration of the above points is provided by the ability of antibiotic-sensitive, and therefore non-super, non-pathogenic bacteria to triumph over a pathogen, *Clostridium difficile*, that is relatively difficult to treat with antibiotics. Intestinal infection with *Clostridium difficile* generally follows treatment with antibiotics but is not reliably eliminated with further antibiotic treatment. A randomized clinical trial [9] performed in Europe compared treatment with 3 regimens: 1) duodenal infusion of feces from screened donors after 4 to 5 days of 500 mg of oral vancomycin 4 times per day with bowel lavage on the last day of antibiotic administration, 2) standard oral vancomycin treatment of 500 mg 4 times per day for 14 days, and 3) oral vancomycin treatment of 500 mg 4 times per day for 14 days plus bowel lavage on day 4 or 5. The regimen including the infusion of feces was much more effective than either regimen with vancomycin but no fecal infusion (over 80% cure rate versus about a 31% or 23% cure rate for vancomycin alone or vancomycin plus bowel lavage).

## HOW TO PROCEED

So, if “super” is a poor choice of prefix to precede “bug” in the context of resistance to multiple antibiotics, how should physicians in general, doctors specializing in infectious disease, microbiologists, journalists, and members of the general public refer to bacterial strains that exhibit resistance to many anti-bacterial drugs. How about referring to multidrug-resistant bacteria as “multidrug-resistant bacteria,” or “therapy-resistant microbes,” or “difficult to treat infectious agents?”

For example, in the literature pertaining to antibiotic-resistant strains of *Mycobacterium tuberculosis* the standard terminology is quite matter-of-fact. *Mycobacterium tuberculosis* is the causative agent of tuberculosis, an infectious disease that remains a major public health threat in many localities around the world and especially for individuals with compromised immune system function. Strains of *M. tuberculosis* that are resistant to multiple therapeutic agents are referred to as “multidrug-resistant” (MDR-TB) and strains that are resistant to all known agents effective against *M. tuberculosis* are labeled “extremely drug-resistant” (XDR-TB). This terminology conveys what is known with certainty without any vague implications that extend way beyond what is known for sure, as is unfortunately the case for the prefix “super.”

As emphasized above, understanding the nature and magnitude of the threat posed by bacterial pathogens is important both for the health of individual patients and the wellbeing of populations, ie, public health. This task can be pursued with maximum success only if the full evolutionary context for pathogen change, spread, and harm is addressed [10]. Medical professionals and journalists alike should recognize that use of imprecise language in the hope of enhancing accessibility does little good if it misinforms instead of educating the intended audience or limits thinking about effective treatments or other interventions. It should not be an excessive burden to explain that multidrug-resistant bacteria are microbes possessing mechanisms that allow them to survive exposure to different antibiotics better than other non-resistant bacteria. Attribution of other properties to bacteria exhibiting resistance to multiple or even all relevant antibiotics by using the prefix “super” is not going to be reliably correct, is therefore unjustified, and is to be strongly discouraged.
